# Long term outcomes of cluster randomized trial to improve cardiovascular health at population level: The Cardiovascular Health Awareness Program (CHAP)

**DOI:** 10.1371/journal.pone.0201802

**Published:** 2018-09-06

**Authors:** Simone Dahrouge, Janusz Kaczorowski, Lisa Dolovich, Michael Paterson, Lehana Thabane, Karen Tu, Jaime Younger, Larry Chambers

**Affiliations:** 1 Department of Family Medicine University of Ottawa, Ottawa, Ontario, Canada; 2 Bruyère Research Institute, Ottawa, Ontario, Canada; 3 Institute for Clinical Evaluative Sciences, Ottawa, Ontario, Canada; 4 Department of Family and Emergency Medicine, Université de Montréal, Montréal, Québec, Canada; 5 Centre de Recherche Center hospitalier de l’Université de Montréal (CRCHUM), Montréal, Québec, Canada; 6 Department of Family Medicine, McMaster University, Hamilton, Ontario, Canada; 7 Leslie Dan Faculty of Pharmacy, University of Toronto, Toronto, Ontario, Canada; 8 Institute for Clinical Evaluative Sciences, Toronto, Ontario, Canada; 9 Institute of Health Policy, Management, and Evaluation, University of Toronto, Toronto, Ontario, Canada; 10 Department of Family and Community Medicine, University of Toronto, Toronto, Ontario, Canada; 11 Western Hospital Family Health Team, University Health Network, Toronto, Ontario, Canada; 12 Department of Health Research Methods, Evidence and Impact, McMaster University, Hamilton, Ontario, Canada; 13 Faculty of Health, York University, Toronto, Ontario, Canada; National Yang-Ming University, TAIWAN

## Abstract

**Study question:**

The Cardiovascular Health Awareness Program (CHAP) cardiovascular risk reduction program consisted of sessions run by local volunteers in local pharmacies during which cardiovascular risk was assessed, healthy lifestyle and preventive care was promoted, and the participants were oriented to local resources to support changes in modifiable risk factors. A clustered randomized trial implemented in September 2006 across 39 communities targeting community-dwelling individuals 65 years and older showed a significant reduction in hospitalization one year after its implementation (rate ratio of 91 [95% confidence interval (CI): 86%-97%]). This study explores the impact of CHAP in the first five years.

**Methods:**

Using health administrative data housed at the Institute for Clinical Evaluative Sciences, we established a closed cohort consisting of all individuals eligible in these communities at the study onset whom we followed over time. We assessed hospitalizations and survival using a negative binomial model for count data and Cox regression to assess time to first event, accounting for the clustered design. The primary outcome was the rate of cardiovascular-related hospitalizations defined as congestive heart failure, stroke or acute myocardial infarction.

**Results:**

Most estimates pointed to an advantage for the intervention arm, but only all-cause mortality reached statistical significance (hazard ratio [95% CI] = 0.955 [0.914–0.999]). The hospitalization cardiovascular-related hospitalization rate ratio was (0.958, 95% CI: 0.898–1.022) in favour of the intervention communities, translating to an estimated 408 averted hospitalizations over the five-year period. There was no evidence of the effect of time from start of intervention.

**Conclusions:**

The consistent direction of the outcomes in favour of the intervention arms suggests that CHAP likely had a meaningful impact on reducing cardiovascular-related morbidity and mortality. Given the low cost of the intervention, further development of CHAP should be pursued.

## Introduction

According to the recently published Global Burden of Disease Study, [1] high blood pressure (BP) is linked to the highest risk of morbidity and premature death worldwide.[2–4] The lifetime residual risk of developing hypertension in adults ages 55 to 65 years is estimated to be 90%,^6^ making high BP a concern for virtually everyone. Effective prevention, early diagnosis and optimal treatment of hypertension can reduce the risk of developing related complications such as heart failure, stroke and other cardiovascular disease (CVD).[5] There are numerous cost-effective pharmacological therapies to lower BP, and there is robust evidence demonstrating that better BP control is associated with significant reductions in morbidity and mortality from CVD as well as all-cause mortality.[6–10] Adopting healthy lifestyle behaviours also significantly contributes to better cardiovascular health. These modifiable risk factors account for 55% of the population attributable risk for CVD, and changes in these behaviours can significantly reduce the risk of the CVD-related outcomes.[10] Extensive and rigorous evidence supports the promotion of maintaining a healthy diet, engaging in regular physical activity, promoting a smoke-free environment, and restricting alcohol and sodium intake in lowering BP and thus preventing associated CVD.[11]

Hypertension is a critical issue especially in seniors, among whom under-diagnosis and under-treatment are most prevalent, and in whom both non-pharmacological and pharmacological treatments are clearly effective at achieving BP targets, and reducing stroke and cardiovascular morbidity and mortality.[12] However, hypertension is frequently undiagnosed, undertreated, and poorly controlled. In Canada, one in six individuals with elevated BP are undiagnosed,[13] and one third of those diagnosed with hypertension do not achieve BP control.[14,15]

While BP management usually takes place in general practice, a more comprehensive approach is needed, especially as far as primary prevention is concerned. The challenges to the conventional office-based primary care approaches include infrequent visits, poor BP measurement techniques, white coat hypertension, masked hypertension, and competing presenting complaints taking precedence over discussions about lifestyle behaviours, self-monitoring or adherence to therapy.[16,17] Effective population-based strategies for health promotion and disease prevention, for people with established CVD and for those at risk of developing it, are seen as critical to counter widespread and growing epidemics of obesity, hypertension, diabetes, heart disease and stroke.[18–21] A recent review of over thirty community-based CVD prevention programs from 1970 to 2008 showed that, while these interventions appear largely effective, a better understanding of their impact is required before further large-scale implementation.[22] Mounting empirical evidence supports the use of community-based programs that involve organizing communities for risk factor screening and supporting patients to make desirable lifestyle changes.[18,23,24] The Cardiovascular Health Awareness Program (CHAP) is one such program (www.CHAPprogram.ca).

In September 2006, our team conducted a pragmatic cluster-randomized controlled trial of CHAP in 39 communities across Ontario with populations between 10,000 and 60,000 targeting individuals 65 years of age and older and residing in the community. An analysis conducted in 2010 revealed a statistically and clinically significant reduction in hospital admissions, in favour of the intervention communities, for acute myocardial infarction, stroke, and congestive heart failure (CHF; composite end point) among all community residents aged 65 and over in the year before compared with the year after implementation of CHAP (rate ratio of 91% [95% confidence interval (CI): 86%-97%]). This translates into approximately 200 averted hospitalizations in the intervention communities.[25] The current study assesses the longer term impact of the CHAP intervention on morbidity and mortality related to cardiovascular events over the five years post intervention.

## Methods

### Design

We conducted a five-year, closed cohort health-administrative-data- based follow-up evaluation of all community-dwelling individuals 65 years of age and older who were eligible for the CHAP cluster randomized pragmatic trial for the period spanning September 1^st^ 2006 through August 31^st^ 2011 (ISRCTN50550004).[26]

### Setting

Thirty-nine Ontario communities with population sizes between 10,000 and 60,000 and that had at least five family physicians and two pharmacies were first stratified according to population size and geographic location (seven strata) to ensure that adjacent communities were not allocated to different arms of the study, and randomly allocated to receive the intervention or control.[25] Ontario, which is Canada’s most populous province with over 13 million individuals, has a universal healthcare system that covers all physician visits and hospitalizations.

### Study arms

Twenty communities were randomly allocated to the intervention arm. We invited all family physicians in these communities to send their eligible patients a letter promoting the program, and inviting them to attend a CHAP session. The program was also broadly publicized within the community. We engaged a local lead organization in each community to take leadership in establishing and championing the intervention program according to the CHAP Implementation Guide.[27] These organizations set up CHAP assessment and information sessions within local pharmacies during which participants with the support of lay volunteers: 1) completed a cardiovascular risk profile; 2) underwent blood pressure measurement using a validated automated blood pressure measuring device; 3) received healthy lifestyle and preventive care promotional materials; and 4) were informed about locally available resources to assist them in addressing modifiable risk factors. Data collected at the CHAP sessions were provided to the participant and also shared with the participant’s family physician and pharmacist to ensure appropriate follow up and information continuity. The program implementation article [27] and full study protocol [28] provide more details on the intervention and the role of volunteers, family physicians and pharmacists in the intervention.

All 20 intervention communities completed the 10-week intervention period beginning September 1^st^ 2006, and held 1,265 three-hour CHAP pharmacy sessions during which 27,359 assessments were performed, including 15,899 unique participants of whom 13,379 individuals were 65 years and older and eligible for the study.[26,29] There were no major cardiovascular initiatives, CHAP or otherwise, in the 19 control communities.[25]

### Data sets

Consistent with the previous analysis,[25] we relied upon routinely collected health administrative data for all publicly insured services funded under the Ontario Health Insurance Plan (OHIP). These datasets are fully anonymized and securely held in encoded form at the Institute for Clinical Evaluative Sciences (ICES, http://www.ices.on.ca). All relevant datasets were linked using unique, encoded identifiers and analyzed at ICES. The 39 communities were defined geographically by postal codes, and their population were identified based on their place of residence.

The study target population was individuals 65 years of age and older who resided in the community, excluding long term care facilities. All residents of the 39 communities meeting the two study eligibility criteria as of the intervention start were identified to form the study cohort. The list was limited to individuals ≥ 65 years of age or older using the OHIP *Registered Persons Database*, and to those residing in the community by excluding residents of long term care facilities identified from the *Continuing Care Reporting System*. The dataset containing 143,976 individuals, 69,318 and 74,658 in the intervention and control communities, respectively, constituted the closed cohort which we followed for five years. The baseline characteristics of that cohort included age, sex and location of residence (Registered Persons Database (RPDB)), chronic conditions (ICES derived disease cohorts for diabetes, CHF and stroke), and medical complexity (using the Adjusted Diagnostic Group). Hospitalization and mortality rates in the previous year were determined as described under outcome.

### Outcomes

In keeping with the original analysis, we considered seven outcomes. The main outcome was CVD-related hospitalizations; a composite endpoint capturing hospitalizations for which the most responsible diagnosis was stroke, CHF, or acute myocardial infarction.[25] For secondary outcomes, we considered the hospitalizations for each condition separately, CVD-related in-hospital deaths and overall, as well as all-cause mortality. We relied on the hospitalization records’ “Most Responsible Diagnoses” in the hospital *Discharge Abstract Database* to establish the reason for hospitalizations and identify deaths occurring during hospitalization,[30–33] and on the *Registrar General—Death* (Vital Stats) to capture cardiovascular-related mortality and all-cause mortality. All analyses were carried out with the intention-to-treat approach, where all 143,976 eligible residents in the trial communities were included in the analyses.

### Statistical analyses

#### Descriptive statistics

We report the baseline demographic profile and outcome rates using proportion and means (standard deviations (SD)) for the communities in each cohort. All other analyses were performed at the individual patient level. Unless otherwise specified, all analyses were planned *a priori*.

We sought to evaluate the cumulative effect of the intervention over the entire five year follow-up period as well as annually throughout that period. We first assessed the effect of the intervention over the five year period. Our primary outcome was the count of CVD-related hospitalizations (Event level) per cumulative 100 person-years follow-up. Secondary outcomes included the rate of hospitalizations for each reason separately, mortality rates, and rates of individuals having at least one hospitalization (Individual level). We planned to apply a Generalized Linear Model with a Poisson distribution function for the count data, using the person-years at risk as the off-set, adjusting for the pre-intervention rate of the event of interest, and clustering at the community level. However, because the models’ deviances were unacceptably large, a negative binomial distribution model was used instead to account for the over dispersion. These regressions were conducted at the individual patient level, using the person follow up time for each community as an offset variable, and accounting for clustering at the community level. We verified the robustness of that approach by repeating the analyses using community level data. In these analyses, each record corresponded to the total events in that community.

We then assessed the effect of the intervention on the primary outcome across the post-intervention years by adding the year as a categorical variable, and treatment*year interaction terms to the equations, and including an autoregressive correlation structure to account for correlation of event totals in each year.

Secondly, we used Kaplan-Meier plots to visually depict the time to first event for each outcome and assessed the hazard of an individual having the outcome of interest over time using a Cox regression model with frailty (random effects) to account for clustering at the community level and adjusting for the pre-intervention baseline rate of the outcome. The proportional hazard assumption was met for all survival analyses.

Finally, because the two groups were found to differ meaningfully in their rurality, we conducted a *post hoc* sensitivity analyses in which that variable was added to the regression equations. Data analyses were conducted using SAS version 9.2 (SAS Institute, Cary, NC). This study was approved by the ICES review board at Sunnybrook Health Sciences Centre, Toronto, Canada; the Bruyere Research Ethics Board, Ottawa, Canada; and the Ottawa Hospital Research Ethics Board, Ottawa, Canada.

## Results

Control communities were somewhat larger than intervention communities (29,114 vs 25,839). The sex, age distribution and morbidity profile of eligible residents was similar in the two groups (Table 1), with control communities having a higher proportion of individuals living in urban areas (15.3% vs 5.1%). In the year prior to the intervention start, the rate of CVD related hospitalizations (Control vs intervention: 2.80% vs 2.88%), deaths during CVD-related hospitalisations (0.44% vs 0.43%), and all-cause mortality (3.43% and 3.53%) amongst individuals meeting the CHAP eligibility criteria were similar in the two groups. Deaths related to CVD appeared slightly lower in the control compared to the intervention communities (0.54% vs 0.60%).

**Table 1:** Baseline profile of communities.

Demographic characteristics (Description of 2006 cohort, as of September 1^st^ 2006)		
	Control	Intervention
Population, all ages (SD)	29,114 (17,035)	25,839 (15,827)
Eligible residents^1^ (SD)	3,892 (2,201)	3,430 (1,862)
Eligible proportion (%)	13.4	13.3
Age, mean (SD)	74.8 (0.45)	74.8 (0.60)
65–74 (%)	52.6	52.6
75–84 (%)	37.0	36.8
85+ (%)	10.4	10.6
Sex (% male)	42.7	43.1
Rurality Index of Ontario (2004)^2^	2004	2004
<10 (%)	15.3	5.1
10 - <45 (%)	67.2	76.3
45+ (%)	17.6	18.6
Morbidity		
Diagnosis of		
Diabetes (%)	22.4	21.3
CHF (%)	10.5	10.7
Stroke (%)	5.3	5.5
ADG ^3^		
Mean (SD)	7.1 (0.2)	7.0 (0.5)
0 (%)	3.1	3.4
1–4 (%)	23.3	24.6
5–9 (%)	48.7	48.0
10+ (%)	25.0	24.0
**Rates/100 individuals (based on the interval September 1**^**st**^ **2005 –August 31**^**st**^ **2006**		
	**Control**	**Intervention**
Hospitalizations (SD)		
Any CVD-Related	2.80 (0.61)	2.88 (0.64)
AMI	0.95 (0.35)	0.95 (0.43)
CHF	1.09 (0.32)	1.09 (0.31)
Stroke	0.76 (0.25)	0.84 (0.15)
Deaths (SD)		
During CVD-Related Hospitalization	0.44 (0.12)	0.43 (0.16)
Related to CVD	0.54 (0.13)	0.60 (0.19)
All-cause mortality	3.43 (0.40)	3.53 (0.58)

This table represents the unweighted average values for each of the 19 control and 20 intervention communities. Each community contributes equally to the estimate, regardless of its population size.

^1^ Number of individuals ages ≥65 years residing in a participating who do not live in long term care.

^2^ The Rurality Index of Ontario was calculated for each community based on the postal code of its residents as of September 1^st^ 2006 according to the 2004 RIO criteria.

^3^ ADG = Adjusted Diagnostic Group. ADG was derived using the Johns Hopkins Adjusted Clinical Groups (ACG) System with which we derived Adjusted Diagnostic Groups from physician claims and hospital admissions using and was based on the two year interval preceding intervention implementation (September 1^st^ 2004 –August 31^st^ 2006).

The 74,658 control and 69,318 intervention individuals (persons (P)) who met the eligibility criteria and were included in the cohort had a total of 337,856 and 315,387 person-years (PY) follow-up duration, respectively (Table 2). In the intervention arm, 13,379 individuals 65 years of age and over, 19% of the eligible individuals, attended at least one CHAP session. During the five year follow up period, 10,565 (3.13 per 100 PY), and 9,304 (2.95 per PY) CVD-hospitalizations, representing 7,852 (10.5%) and 6,978 (10.1%) individuals, were documented in the control and intervention communities, respectively. Death during CVD-related hospitalization, cardiovascular related mortality, and any cause mortality was 2.2% vs 2.0%, 3.0% vs 2.8%, and 19.6% vs 18.9%, of individuals in the control and intervention communities, respectively.

**Table 2:** Five year cumulative rates (September 1^st^ 2006—August 31^st^ 2011).

	**Control**		**Intervention**	
Cohort size (all eligible individuals)	74,658		69,318	
Person-Years follow-up	337,856		315,387	
				
Outcomes–**Event** level (#/100PY)	**Numbers**	**Rates/100 PY**	**Numbers **	**Rates/100 PY**
Hospitalizations				
Composite–Any CVD-Related	10,565	3.13	9,304	2.95
*AMI*	*3*,*560*	*1*.*05*	*2*,*958*	*0*.*94*
*CHF*	*4*,*233*	*1*.*25*	*3*,*798*	*1*.*20*
*Stroke*	*2*,*772*	*0*.*82*	*2*,*548*	*0*.*81*
Outcomes–**Individual** level (#/100P)	**Numbers**	**Rates/100 P**	**Numbers **	**Rates/100 P**
Hospitalizations				
Composite–Any CVD-Related	7,852	10.5	6,978	10.1
*AMI*	*3*,*139*	*4*.*2*	*2*,*594*	*3*.*7*
*CHF*	*2*,*922*	*3*.*9*	*2*,*667*	*3*.*8*
*Stroke*	*2*,*531*	*3*.*4*	*2*,*346*	*3*.*4*
Deaths				
During CVD-Related Hospitalization	1,639	2.2	1,394	2.0
Related to CVD	2,259	3.0	1,968	2.8
All-cause mortality	14,641	19.6	13,103	18.9

**Fig 1:**
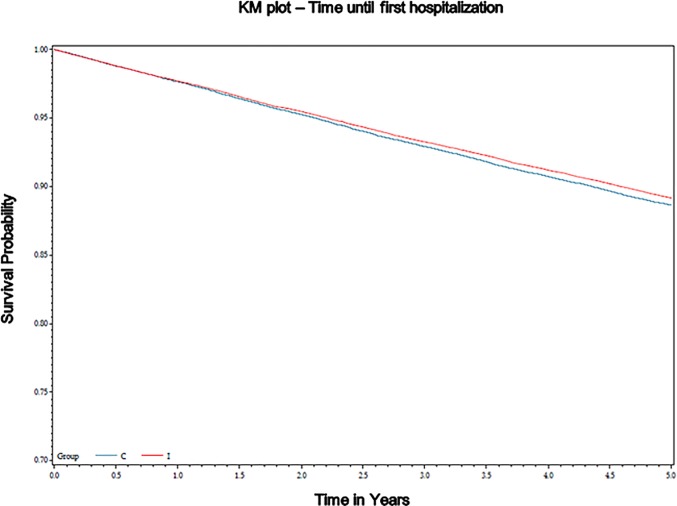
Time to first event for each outcome. Kaplan Meier curves represent the time to first event for each individual in the intervention and control communities. These graphs do not account for the clustered design of the study.

The Kaplan Meier plots for these events are show in Fig 1.

**Fig 2:**
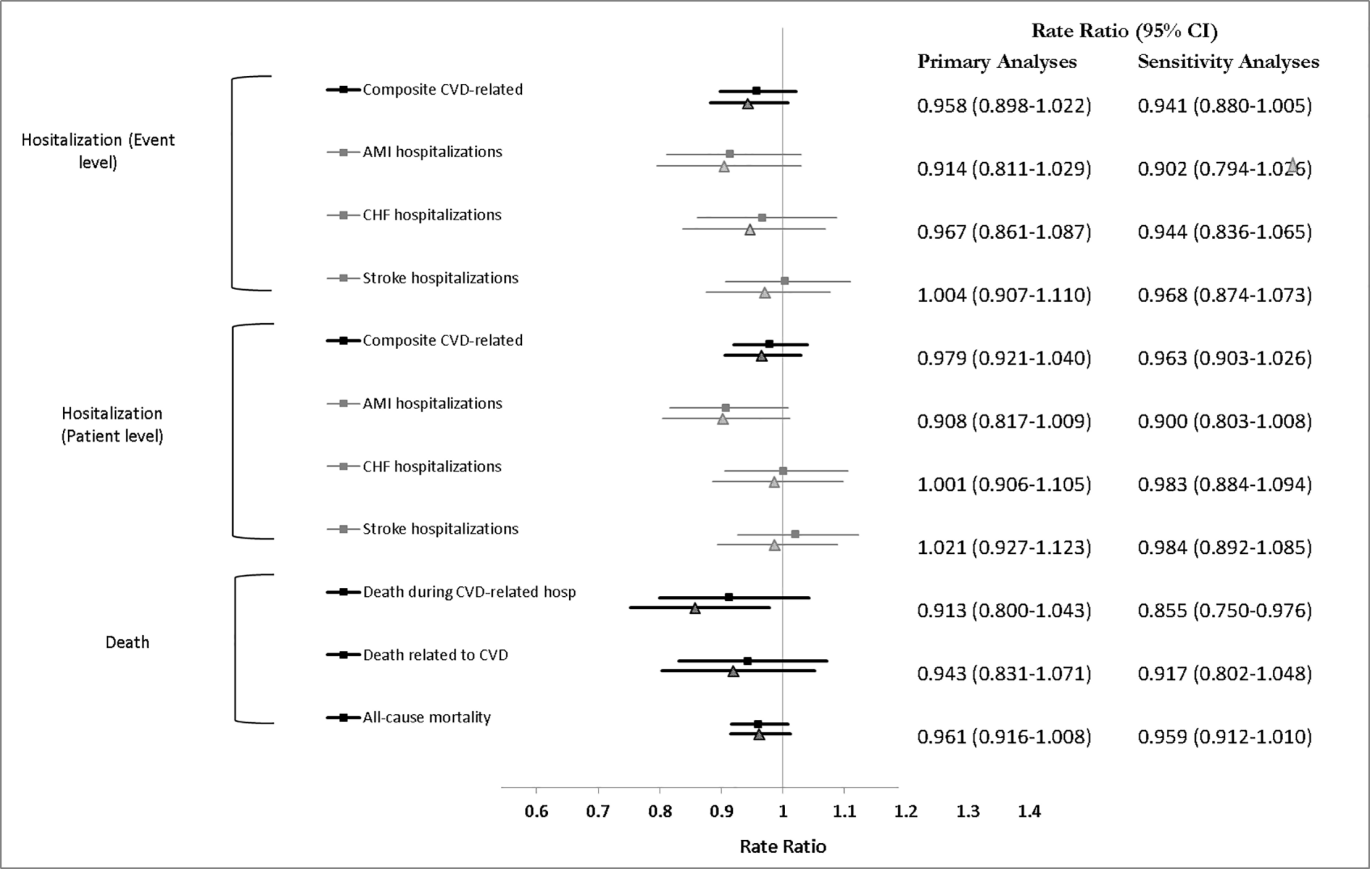
Forest plot showing the results of the negative binomials rate ratios and Cox (hazard ratios) regressions. Primary analyses are results of regressions established *a priori* and in which the baseline rate for that event is used as the offset (Rate Ratio) or as a covariate in the model (Hazard Ratio), and where the clustering of individuals within the community is accounted for. Sensitivity analyses also included the rurality category (<10, 10–45, >45). These were planned *a posteriori*, after identifying a meaningful difference in the rurality index of the two groups of communities. Cox regressions were conducted for “Patient level” outcomes only, while negative binomial regressions were carried out for both “Patient level” and “Event level” outcomes. All measures reflect the rate of the event in the intervention communities compared to the control communities. An estimate smaller than 1 points to a benefit for the intervention arm.

The planned Generalized Linear Model with a Poisson distribution function produced unacceptably large dispersion values, ranging from 3.0 to 6.1, across all outcome measures. The negative binomial analyses we applied produced very similar results when conducted at the patient and community level. For example, for the composite measure of cardiovascular hospitalization, the estimated intervention effect and standard error were -0.0429, and 0.0328, respectively. The *p* values were 0.191, and 0.199, respectively. We report on the results of the patient level negative binomial analyses and the *post-hoc* sensitivity analyses in which rurality was added to the equation to account for the imbalances observed (Fig 2).

The five-year cumulative rate ratio [95% confidence interval (CI)] for CVD-related hospitalizations (main outcome) was 0.958 [0.898–1.022]. This translates into an estimated 408 averted hospitalizations, or 5.9 per 1,000 individuals over the five-year period. Considering both analytical approaches, most estimates pointed to an advantage for individuals residing in the intervention communities, but only the hazard ratio for all-cause mortality was significantly different across the groups (Hazard ratio [CI] = 0.955 [0.914–0.999], *p* = 0.0429). Accounting for the rurality status of the individuals in the community in the post hoc analyses, the rate (ratio: 0.855 [0.750–0.976]) and hazard (ratio: 0.862 [0.770–0.965]) of death occurring during CVD-related hospitalizations were statistically significantly in favour of the intervention arm.

The autoregressive model did not reveal any significant differences in intervention impact in any years across the four cardiovascular hospitalization count measures (*p* values for the composite score and individual reasons for hospitalization ranged from 0.20 and 0.94). Given this, applying the model to secondary outcome measures was not warranted.

## Discussion

We found a consistent pattern favouring better health outcomes in the intervention compared to the control communities, with all-cause mortality reaching statistical significance. While the observed effect sizes are generally relatively small, because these cover a five-year span, the potential of yielding meaningful clinical and health system benefits exists. These findings extend those observed in our initial analysis of the trial, which showed statistically significant reductions favouring the intervention communities in the risk of cardiovascular-related hospitalizations in the second year after the start of the intervention (rate ratio: 0.91 [0.86 to 0.97]).[25] In the present analysis, we estimated the five-year cumulative rate ratio to be 0.96 (0.90–1.02), without clear evidence of differences across the years.

CHAP aimed to reduce the risk of poor outcomes associated with hypertension and other modifiable risk factors. Based on the Wagner’s Chronic Care Model, CHAP is a multipronged strategy to address cardiovascular risk factors. CHAP seeks to inform and empower patients to take action on cardiovascular healthy behaviour and to support health care delivery.[34] It offers community-based cardiovascular screening and education sessions that include promoting access to community resources that can help address individuals’ health needs. CHAP also supports information continuity and linkages between community care and primary health care professionals. This system-level intervention relies on a multipronged approach which includes individual patient and care provider components, and is expected to lead to reduced risk of poor cardiovascular outcomes through increased healthy behaviour and improvements in health care delivery, including hypertension pharmacotherapy.[35,36] The latter was documented in an earlier analysis that demonstrated a 10% increase in the rate of newly prescribed antihypertension treatment in the year following the intervention.[25]

Survival benefits associated with adequate blood pressure control can be observed shortly after control is achieved, and continue to be manifested over many years.[37,38] Similarly, changes in lifestyle factors, such as smoking cessation [39] or exercise [40], can also be expected to produce survival benefits over a prolonged period of time. All information collected during CHAP sessions was communicated to the individual’s primary care provider with the expectation that abnormal blood pressure findings and health risk behaviour would be addressed. Our previous analyses suggested that the intervention likely allowed previously undiagnosed hypertensive patients to be identified. The one-year post intervention analysis demonstrated a 10% increase in the likelihood of patients residing in the intervention communities initiating anti-hypertensive therapy compared to those in the control communities, likely reflecting response to a higher hypertension detection rate in these communities. It is also expected that patients with known hypertension who had blood pressure measurements outside the target range may have had a change in medication management to improve control, both of which would be expected to translate into a reduced risk of poor outcome.[37,38] The healthy lifestyle behaviour risk assessment performed during the CHAP sessions was used to focus the discussion between the volunteer and participant about the risks associated with such behaviour, and the potential benefit of community resources that support individuals achieve lifestyle goals and how these can be accessed. Primary care providers may also have supported and reinforced the recommendations for actions on modifiable risk factors at subsequent visits. Evidence supports the benefit of such programs in improving self-efficacy and achieving lifestyle goals [9,41–46] and their health benefits.[39,40] Community health promoting services, such as the resources to which participants were directed during the CHAP sessions, play an important role in supporting individuals to achieve their health goals. The U.S. Preventive Services Task Force Recommendation Statement strongly recommends that individuals with known CVD risk factors be provided education and support to overcome risks associated with a poor diet and a sedentary lifestyle.[47] Similarly the National Guidelines for Diabetes Management highlight the role self-management education and support programs provide in the community in achieving healthy outcomes.[48] In addition, a recent systematic review found strong evidence supporting the use of peer-led community-based educational programs in improving outcomes amongst individuals with chronic conditions.[46] This study provides additional evidence of the benefit of peer-led support and educational resources in improving patient outcomes.

The results of this study suggest an apparent reduction in morbidity and mortality in the intervention arm over the five-year period, with no evident attenuation of the observed effect over time. These initial analyses demonstrated a 9% reduction in the risk of CVD hospitalization in the first year following the intervention year. The present study suggests an average 4% [-2%-10%] risk reduction in the five years following the intervention: a lower yearly estimate, but potentially prolonged clinically meaningful consequences.

Despite significant investments in recruitment efforts, only approximately one in five eligible individuals attended at least one CHAP session during the 10-week intervention period, thus limiting the potential to produce a broad impact at the population level. However, because CHAP is a low-intensity intervention, most intervention communities (16 of the original 20) continued to offer some elements of CHAP for several years. Most offered the CHAP pharmacy sessions at weekly or twice monthly intervals during which blood pressure monitoring and recommendations to access health-enabling resources in the community were provided. However, a crucial component, the linkage to primary health care providers, proved more challenging and was dropped by virtually all communities. This ongoing investment in some CHAP elements amongst intervention communities may have contributed to the apparent sustained effect.

The risk protection observed in the CHAP communities was especially compelling for survival. In-hospital death related to cardiovascular disease was lower in the intervention communities, and the effect estimate was more apparent when the model accounted for the imbalance in rurality across the two groups. The potential effect of the intervention on a broad range of health risks that are compounded by an unhealthy lifestyle [46,49] has contributed to the study’s ability to detect statistically significant survival benefits. However, we cannot exclude the possibility that chance alone accounted for the observed difference.

Morbidity and mortality related to cardiovascular disease is significant [50,51] and a very important proportion of this burden can be prevented because many of the risk factors leading to cardiovascular disease and its poor outcomes are known and modifiable.[35,36] Multipronged strategies, including population-based efforts that target individuals and health care providers, such as CHAP, are required to address these modifiable risk factors.

CHAP is a relatively simple and effective patient-centered program that can be established and maintained in small communities. CHAP implementation can be tailored to accommodate the profile of the community in which it is embedded. It facilitates the coordination and integration of primary care and community services. Because the program is largely volunteer peer led, relies on existing community resources for its operation, and requires minimal infrastructure investment to establish, its costs are low. A 2013 evaluation demonstrated it to be cost effective.[52] Our team continues to develop the CHAP program and adapt the approach to address the specific conditions of various populations.

### Limitations

Our study had both strengths and limitations. It was a randomized controlled trial with, but for provincial emigration, no loss to follow-up. Outcome assessment relied on validated, clinically relevant measures available for all individuals.

However, because the study relied exclusively on health administration data, we did not have access to information that could help elucidate the findings. For example, we were unable to measure the participants’ access to the recommended resources, nor assess changes in patient health behaviours, blood pressure levels, or other factors that could have helped explain the pathway through which the intervention imparts cardiovascular protection. We also did not have data on the intensity with which the intervention communities maintained CHAP activities and how these might have been adapted to help understand the impact of these factors on the intervention five-year effectiveness measures. There were also too few communities to assess whether some community attributes, such as extent of community resources available to participants, may have influenced the likelihood to benefit from the intervention.

A number of factors could have reduced our ability to detect differences between the arms. For example, the closed cohort approach does not account for migration of individuals across communities over time. The increased awareness of cardiovascular disease could potentially have led to an increased likelihood of assigning cardiovascular disease as a reason for hospitalization. The small number of clusters in the study, and the statistical approach required may also have impeded the study power. The scaled deviances values, which ranged from 3 to 6, pointed to overdispersion in the outcome that required an alternative model be applied. The negative binomial distribution we used produced similar effect size estimates, but considerably higher variance, and therefore broader confidence intervals.

The CHAP model mobilizes existing resources, connecting community services, family physicians, pharmacists and volunteers in the effort to address modifiable CVD risk factors, many of which play an important role in the aetiology of numerous chronic conditions and could have broad benefits.[53] Given the low cost of the intervention, further development of CHAP should be pursued.

## Conclusions

The results of these analyses covering a five-year period build on the previous short-term analyses that demonstrated a benefit of the CHAP intervention in reducing the risk of CVD-related hospitalization. While the present results point to a more favourable outcome in communities assigned to the intervention arm, this five-year effect size was smaller, and the study lacked power to reach statistical significance.
